# *Urtica* spp.: Ordinary Plants with Extraordinary Properties

**DOI:** 10.3390/molecules23071664

**Published:** 2018-07-09

**Authors:** Dorota Kregiel, Ewelina Pawlikowska, Hubert Antolak

**Affiliations:** Institute of Fermentation Technology and Microbiology, Faculty of Biotechnology and Food Science, Lodz University of Technology, 171/173 Wolczanska, 90-924 Lodz, Poland; dorota.kregiel@p.lodz.pl (D.K.); ewelina.pawlikowska@edu.p.lodz.pl (E.P.)

**Keywords:** *Urtica* spp., bioactive compounds, antioxidant activity, antimicrobial activity, traditional medicine, food industry, animal breeding

## Abstract

Nettles (genus *Urtica*, family Urticaceae) are of considerable interest as preservatives in foods for both human and animal consumption. They have also been used for centuries in traditional medicine. This paper reviews the properties of nettles that make them suitable for wider applications in the food and pharmaceutical industries. Nettles contain a significant number of biologically-active compounds. For example, the leaves are rich sources of terpenoids, carotenoids and fatty acids, as well as of various essential amino acids, chlorophyll, vitamins, tannins, carbohydrates, sterols, polysaccharides, isolectins and minerals. Extracts from the aerial parts of nettles are rich sources of polyphenols, while the roots contain oleanol acid, sterols and steryl glycosides. Due to the variety of phytochemicals and their proportions they contain, nettles show noticeable activity against both Gram-positive and Gram-negative bacteria. These properties make nettles suitable for a range of possible applications, including functional food, dietary supplements and pharmacological formulations. Despite these benefits, the nettle is still an underestimated plant source. This paper provides a unique overview of the latest research on nettle plants focusing on the possibilities for transforming a common weed into a commercial plant with a wide range of applications. Special attention is paid to the antimicrobial activity of the active compounds in nettles and to possible uses of these valuable plants in food and feed formulations.

## 1. Habitats of *Urtica* spp. Plants

The genus *Urtica* belongs to the family Urticaceae in the major group Angiosperms (flowering plants). There are 46 species of flowering plant of the genus *Urtica* [1] (Table 1). The most prominent members of the genus are the stinging nettle *Urtica dioica* L. and the small nettle *U. urens* L., which are native to Europe, Africa, Asia and North America. Plants belonging to the genus *Urtica* are herbaceous perennials and can grow up to 2 m tall. Serrated leaves are attached in pairs opposite each other to the stem. The soft leaves and the rest of the plant are coated in hairs, some of which sting. The serrated, hairy leaves and sting are generally recognized characteristics of this plant [2]. However, the European variety *U. galeopsifolia* does not have stinging hairs [3]. The underground roots by which the plant spreads are noticeably yellow. Small flowers, each with four greenish-white petals, sit in dense clusters on elongated inflorescences towards the top of the stem.

The word “nettle” is said to derive from the Anglo-Saxon word “noedl” meaning “needle”, while its Latin name “urtica” means “to burn”. This refers to the stinging effects of the tiny hairs on the stems and leaves, which when rubbed against the skin cause a burning sensation and temporary rash. The hairs, so small they are almost invisible to the naked eye, are referred to as “trichomes”. When touched by the skin, the bulbous tips of the trichomes break off, leaving sharp, needle-like tubes [4]. These can pierce the skin and inject a fluid-containing substances including formic acid, the same chemical in ant and bee stings. Other compounds contained in the fluid include histamine, acetylcholine and serotonin. When these toxins are delivered into the skin, a painful itching and burning sensation occurs that may last up to 12 h. The hairs are naturally designed to protect the plant from insects.

Nettles grow all over the world in mild to temperate climates. They prefer open or partly shady habitats with plenty of moisture and are often found in forests, by rivers or streams and on roadsides. *Urtica* spp. are widespread throughout Europe and North America, North Africa and in parts of Asia. Both species of stinging nettle (*U. dioica* L. and *U. urens* L.) prefer to grow in nitrogen-rich soil and are commonly found in soils high in inorganic nitrates and heavy metals. Heavy metals are poorly processed by the plant and tend to accumulate in the leaves. Long vegetation seasons lead to on-going growth, while harsh winters cause destruction of the plants [5].

Nettles are considered weeds due to their rapid growth and soil coverage. However, there are economic and ecological reasons for cultivating stinging nettles. According to Dreyer and Müssing, nettles can improve soils over-fertilized with nitrogen and phosphate [5]. They can also promote the biodiversity of local flora and fauna [6,7]. Over 40 species of insect are supported by nettles [8]. *U. dioica* can reduce heavy metal content in soil [9]. *Urtica* spp. can be used to produce new high-quality agricultural raw materials for the dyeing, textile and energy sectors [7]. Due to their content of tough fibers, nettles were used in Germany and Austria to make textiles during the First World War [10]. Despite these benefits, most stinging nettles are wild harvested. Primary producers of stinging nettles include Eastern Germany, the former USSR, Bulgaria, the former Yugoslavia, Hungary and Albania [5].

## 2. Phytochemical Composition of *Urtica* spp.

Different factors affect the chemical composition of nettle plants, such as the variety, genotype, climate, soil, vegetative stage, harvest time, storage, processing and treatment [11,12,13]. Stinging nettles are a rich source of nutrients. A comprehensive proximate analysis showed that harvested upgrowths contained approximately 90% moisture, up to 3.7% proteins, 0.6% fat, 2.1% ash, 6.4% dietary fiber and 7.1% carbohydrates [10]. On the other hand, nettle leaf powders contain on average 30% proteins, 4% fats, 40% non-nitrogen compounds, 10% fiber and 15% ash (Table 2).

In a study of nettles by Rafajlovska et al., higher quantities of proteins were found in the leaves than in the stems and roots. The content of proteins in the leaves ranged from 16.08 ± 0.38–26.89 ± 0.39%, depending on the source of the sample. The highest protein contents in the stem and roots were 14.54 ± 0.27% and 10.89 ± 0.11%, respectively [14]. Other studies of nettle composition have found that the plants contain a significant number of biologically-active compounds. The nettle leaves contain terpenoids [15], carotenoids [16] including β-carotene, neoxanthin, violaxanthin, lutein and lycopene [17,18], fatty acids, especially palmitic, *cis*-9,12-linoleic and α-linolenic acids [17,19], different polyphenolic compounds [20,21,22,23], essential amino acids, chlorophyll, vitamins, tannins, carbohydrates, sterols, polysaccharides, isolectins [16,17,24] and minerals [25,26], the most important of which is iron.

The leaves of *Urtica* spp. contain around 4.8 mg/g DM of chlorophyll, depending on the climate and environmental conditions. Interestingly, more chlorophyll and carotenoids are usually found in plants that have been harvested from shady places. Kukrić and co-workers noted that there were differences in the content of chlorophyll and carotenoids in leaves of different ages [16]. The concentration of chlorophyll increases in growing leaves and decreases during plant aging. The fresh leaves contain high concentrations of vitamins A, C, D, E, F, K and P, as well as of vitamin B-complexes [23]. The leaves are also known to contain particularly large amounts of the metals selenium, zinc, iron and magnesium. Rafajlovska et al. noted that stinging nettle leaves, stems and roots contained larger amounts of calcium than magnesium. These two elements were present at quantities almost three-times higher in the leaves than in the stems and roots. The calcium content expressed in relation to the dry mass ranged from 2.63–5.09% in leaves, from 0.76–1.42% in stems and from 0.61–0.92% in roots. Zinc was found in the highest concentrations in the leaves (27.44 mg/kg of dry mass), followed by copper (17.47 mg/kg) and manganese (17.17 mg/kg). The mean values for cobalt content were significantly higher in leaves than in stems and roots. The contents of cobalt in the leaves, stems and roots, respectively, were in the ranges of 0.11–0.21 mg/kg, 0.10–0.18 mg/kg, and 0.08–0.16 mg/kg, relative to the corresponding dry mass. *Urtica* leaves in addition contain boron, sodium, iodine, chromium, copper and sulfur [14].

The total phenolic content of one gram of nettle powder has been reported as 129 mg GAE (Gaelic Acid Equivalent), which is two-times higher than the phenolic content in 100 mL of cranberry juice (66.61 mg GAE) [27]. Stinging nettles have been shown to be richer in individual polyphenols than other wild plants [13]. Ghaima and co-workers found that the content of phenolic compounds in stinging nettle leaves was significantly higher than in dandelion leaves [28]. Vajić et al. reported that the predominant phenolic compound in stinging nettle leaves is rutin [29]. Ðurović and co-workers studied the chemical composition of stinging nettle leaves using different analytical approaches. Soxhlet extraction was performed and qualitative analysis of Ultrasound-Assisted (UA) extracts using the UHPLC-DAD technique with MS/MS. Differences in the chemical profiles were found. For example, after Soxhlet extraction, syringic, cinnamic and protocatechuic acids were detected in the products, which was not the case with the UA extract. On the other hand, ferulic, caffeic, chlorogenic and sinapic acids were detected only after ultrasound-assisted extraction [30].

Orčić and co-workers quantified various plant phenolics in methanol extracts of *U. dioica*, for flowers, roots, stems and leaves separately, harvested from different locations in Serbia [20]. The polyphenol profiles were dependent not only on the parts of the plants, but also on the location of their acquisition. The researchers found that inflorescence extracts were the richest in phenolics. The most abundant compound was 5-*O*-caffeoylquinic acid (chlorogenic acid), followed by quercetin 3-*O*-rhamnosylglucoside (rutin) and 3-*O*-glucoside (isoquercetin).

The quantitative and qualitative composition of the roots differs from that of the aerial parts of the plants [22]. The content of the majority of the phenols in the root extracts is significantly lower, and the only prominent compound is secoisolariciresinol. Therefore, the roots of stinging nettles are considered to be the poorest part in terms of bioactive compounds. The root extracts contain only a few acids and derivatives in significant amounts (Table 3) [20]. They contain starch, gum, albumen, sugars and resins, as well as neurotransmitters and receptors, such as histamine, acetylcholine, choline or serotonin. Methanolic extracts of nettle roots have an inhibitory effect on aromatase, a key enzyme in the biosynthesis of estrogens. The lipophilic fractions usually contain phytosterols, pentacyclic triterpenoids, coumarins, ceramides and hydroxyl fatty acids. In turn, isolectins and some polysaccharides have been isolated from the hydrophilic fractions [31]. Krauss and Spitteler identified eighteen phenolic compounds (including homovanillyl alcohol, vanillin, vanillic acid and phenylpropanes) and nineteen ligands (including isolaric, iresinol, secoisolariciresinol and neoolivil) in root extracts [32]. Scopoletin, a coumarin derivative, has also been identified in nettle roots [24]. All these compounds are considered to be very important in medicine and pharmacology. For example, homovanillyl alcohol has been shown to protect against cardiovascular disease [33], while histamine influences the complex physiology of brain systems, affecting cognitive processes, including learning and memory [34], as well as neurotransmitters involved in neuromodulation processes [35]. Phytosterols reduce the absorption of cholesterol in the gut and thereby lower blood cholesterol levels [36]. Scopoletin is a stimulator of lipoprotein lipase activity and protects against cardiovascular diseases [37]. Lignans improve immune responses [38].

Fresh nettle leaves contain smaller amounts of sterols and higher concentrations of flavonol glycosides. The leaves of the plant also contain carotenoids, mainly β-carotene, violaxanthin, xanthophylls, zeaxanthin, luteoxanthin and lutein epoxide [5]. Terpene diols, terpene diol glucosides, α-tocopherol, as well as five monoterpenoid components have also been detected in nettle leaves [39]. Weglarz and Roslon studied the content of polyphenolic acids in leaves and rhizomes. They found that the level of these compounds was higher in the male forms, but the chemical profiles of polyphenolic acids from the female plants were much more diverse [40,41]. Moreover *U. dioica* is considered the only plant that contains choline acetyl-transferase, an acetylcholine-synthesizing enzyme [42]. Fifteen hydroxycinnamic acid derivatives and sixteen flavonoids, flavones and flavonol-type glycosides were identified in hydroalcoholic extracts from the aerial parts of *U. dioica*, *U. urens* and *U. membranacea* using HPLC-PDA-ESI/MS. Of these, 4-caffeoyl-5-*p*-coumaroylquinic acid and three statin-like 3-hydroxy-3-methylglutaroyl flavone derivatives were identified for the first time in *U. urens* and *U. membranacea*, respectively. *U. membranacea* showed a higher content of flavonoids, mainly luteolin and apigenin glycosides, which are almost absent in the other species studied [43].

The hairs of *Urtica* plants contain an acrid fluid with the active components: acetylcholine, histamine and formic acid, as well as silica, serotonin and 5-hydroxy tryptamine. Many of these chemicals are smooth muscle stimulants [44]. The fresh hairs of *U*. *dioica* also contain a high level of acetylcholine [45]. The results of numerous experiments suggest that each species of nettle, as well as each part of the plant (root, stalk or leaves) have a different content and profile of bioactive compounds. Therefore, different species of nettle may have different uses, depending on their chemical characteristics [21].

Generally, the phenolic composition of plants is affected by different factors, including the variety, genotype, climate, soil, vegetative stage of the plant, harvest time, storage, processing and treatment [46,47]. When and how nettles are harvested strongly determines the final product. For example, for fiber production, stinging nettles should be harvested when the seeds are mature or when the stalks reach 80% of the aboveground biomass, from the second year of planting. During the first year, the stalks are too thin, too ramified and have too many leaves. If the main product is to be the leaves, younger plants are harvested. The time of year for nettle harvesting depends on the purpose. Plants collected in April are used for fodder, medicine or chlorophyll production. Nettles harvested at the end of June are used for fiber production. The second harvest in September may be used for the collection of leaves [7].

## 3. Antimicrobial Activities of *Urtica* spp.

Nettles possess noticeable antimicrobial activity against Gram-positive and Gram-negative bacteria when compared with standard and strong antimicrobial compounds, such as miconazole nitrate, amoxicillin-clavulanic acid, ofloxacin and netilmicin [48]. Different fractions of various *Urtica* species have been studied to determine their antimicrobial activity. The results indicate the great potential of this plant for the discovery of novel effective compounds (Table 4 and Table 5) [49,50,51,52,53,54,55,56].

The results presented in Table 5 show the antimicrobial activities of various nettle extracts obtained by different researchers. As can be seen, some nettle extracts show activity at a concentration of 72 mg/mL and others at 1 μg/mL. These differences appear excessive, and the results should therefore be viewed with caution. Such variations may be associated with the location of the plant habitat and climactic conditions, as well as being due to the use of different extraction techniques and evaluation methods. Despite their significant differences, however, the results of these studies show that nettle plants exhibit antimicrobial activity against a wide spectrum of microbial strains, often isolated from foods of low microbiological quality. A study by Kukrić et al. revealed that nettle extracts had inhibitory effects on various Gram-positive and Gram-negative bacteria including *Bacillus subtilis*, *Lactobacillus plantarum*, *Pseudomonas aeruginosa* and *Escherichia coli* [16]. Mahmoudi et al. reported that all microorganisms tested in their research, Gram-negative and Gram-positive bacteria, as well as *Candida albicans* yeast, were sensitive to alcoholic extract from the nettle stem [62]. In recent studies conducted by Antolak et al., the ethanol extract of *U. dioica* showed inhibitory activity against the growth of acetic acid bacteria belonging to the genus *Asaia*, a beverage spoilage bacteria found in functional drinks [63]. On the other hand, Shale et al. noted that *E. coli* and *P. aeruginosa* were completely resistant to the ethanol and methanol extracts from stems and leaves of *U. dioica* [64]. Different antimicrobial properties may be the result of the isolation of different compounds in different solvents, of different extraction efficiencies and possibly of chemical degradation by polar and non-polar solvents. The extraction method, the plant type, the geographical and ecological status, the climate, seasonal and experimental conditions, the age of the plant, environmental stress factors, as well as inter-species differences all play a role and may explain the diversity of results in different studies [21,65,66].

## 4. *Urtica* spp. in Traditional and Modern Medicine

Nettles are one of the most commonly-used medicinal plants in the world, due to their health-enhancing qualities. Because of their high content of nutritive substances, nettles are also used in folk veterinary medicine [48,67,68]. There are many dietary supplements based on *Urtica* spp. now on the market. Their popularity can be explained by their non-toxic chemical composition, relatively low cost and wide availability. The most recognized health benefit of using stinging nettles is activity against Benign Prostatic Hyperplasia (BPH), also known as an enlarged prostate, as well as urinary tract infections. Clinical studies suggest that *Urtica* spp. contain compounds that affect the hormones responsible for BPH. In addition, nettle root extract shows activity against prostate cancer cells. In therapy, nettles are usually used in combination with saw palmetto (*Serenoa repens*) [69,70]. They are also used as a home remedy for bladder infections.

Nettles can help alleviate the symptoms of osteoarthritis and joint pain, typically in the case of hands, knees, hips and spine. Nettles can work in combination with nonsteroidal anti-inflammatory drugs (NSAIDs), allowing patients to decrease their use of NSAIDs. The prolonged use of NSAIDs can increase the risk of heart attack or stroke. In a study by Randall and co-workers, nettles were able to decrease osteoarthritic pain in the base of the thumb when applied to the painful area. In a clinical trial of 37 people with acute arthritis, 50 g of stewed nettle leaves consumed daily, combined with 50 mg of diclofenac, were shown to be as effective as the full 200-mg dose of diclofenac over a two-week period [71]. Studies have also shown that applying nettle leaves directly decreases joint pain and can treat arthritis. In a study by Christensen and Bliddal, it was found that a combination of nettles, fish oil and vitamin E reduced the need for analgesics and other drugs for the symptoms of osteoarthritis [72].

Another study conducted by Klingelhoefer et al. showed the anti-inflammatory benefits of stinging nettles against other autoimmune diseases, such as rheumatoid arthritis [73]. Nettle leaves contain histamine, which may seem inadvisable for allergy medication. However, histamine has been already used to treat strong allergy symptoms [74]. Histamine production causes unwanted allergic reactions, associated with unpleasant nasal congestion, sneezing or itching. Stinging nettles affect numerous receptors and/or enzymes involved in allergic reactions [75]. In addition, because of their anti-histamine and anti-inflammatory properties, stinging nettles can be used as a natural component in eczema medications. Infusions of the plant can be used for nasal and menstrual hemorrhage, diabetes, anemia, asthma, hair loss and to promote lactation [76]. Terpenes and phenols are major groups associated with the inhibition of cancers, as well as with the treatment of headache, rheumatism and some skin diseases [58,77,78]. Phenols also have been associated with the inhibition of atherosclerosis and cancer, as well as age-related degenerative brain disorders [79,80].

The combination of *U. dioica* with common thyme (*Thymus vulgaris*), liquorice (*Glycyrrhiza glabra*), common grape (*Vitis vinifera*) and lesser galangal (*Alpinia officinarum*) has been known in Turkey as an Ankaferd Blood Stopper (ABS). This traditional medicine works on endothelium, blood cells, angiogenesis, cellular proliferation, vascular dynamics and cell mediators to stop bleeding [81]. In a study conducted by Bourgeois et al., nettles were used for cosmetic applications as an anti-aging complex, involving the inhibition of collagenase and elastase activities. These properties could be ascribed to the ursolic acid and quercetin present in the nettle extracts [82].

Herb extract of *Urtica* plants is useful for bladder disorders, reduces postoperative blood loss and prevents hemorrhagic and purulent inflammation following adenomectomy. Aqueous infusions of *U. dioica* exhibit antioxidant activity towards iron-promoted oxidation of phospholipids, linoleic acid and deoxyribose [83]. For a long time, the hypoglycemic effects of *U. dioica* were only speculative. Recent studies show that nettles possess anti-diabetic properties [84]. Thus, nettles could serve as good adjuvant to other oral hypoglycemic agents and seem promising for the development of phytomedicines for diabetes mellitus. In addition, as organic nitrogenous compounds, amino acids from nettles are building blocks in the process of protein biosynthesis [85]. The safety of aqueous extracts of *U. dioica* and their antidiabetic effects have been confirmed with mice models [86].

## 5. Food and Feed Applications

Nettles have traditionally been used as a nutritious food, particularly in spring time in rural areas. The Romans are said to have consumed nettles, and a recipe for Saint Columba’s broth survives to this day. In Greek and Roman times, nettle roots were used for meat tenderization. Nowadays, nettles are used in a large number of recipes. *Urtica* spp. are blended with fromage blanc, potatoes and nutmeg to make nettle nouvelle [39]. Stinging nettles remain popular, especially in poor countries and among the lower socioeconomic classes [87]. For example, amino acids from dehydrated nettle meal are nutritionally better in comparison to those of alfalfa meal [88]. These plants may be consumed primarily as a boiled or cooked fresh vegetable, which is added to soups, cooked as a pot herb or used in vegetable salads [10]. In the temperate region of the Himalayas, *U. plaviflora* leaves are cooked and eaten as a green vegetable. Upgrowths and leaves are collected with the help of bamboo or iron pincers and cooked as soup. The plants are boiled with maize, millet or wheat flour, with the addition of salt and chili to make a kind of porridge. Due to their seasonality, *U. plaviflora* plants are preserved by lactic acid fermentation [4].

In European countries, nettles are used in soup or as a steamed or wilted vegetable. Since it has a similar flavor and texture, cooked nettle can be used as a substitute for spinach. Raw nettles after blending can be also used in pesto sauces, salad dressings or dips. Boiled nettles with walnuts is a common dish in Georgia, while Romanians make sour soup using fermented wheat bran, vegetables and young nettle leaves [89]. Mature leaves are used in the production of semi-hard Cornish cheese, made from grass-rich milk and wrapped in stinging nettles. The nettle changes the acidity of the outside of the cheese, affecting the way the curd breaks down and matures. It has also been documented that nettle leaves can be used to coagulate milk in the process of fresh cheese making [90]. In some European countries, for example in Serbia and Poland, bread with nettle leaves (up to 1%) is sold as a commercial product [30]. Compared to barley and wheat flour, nettle flour has a much higher content of proteins, crude fibers, fats, ash, calcium and iron and has a low glycemic index. As compared to barley and wheat, nettles have much higher levels of tannins and total polyphenols [10].

Nettle leaves can also be used to make an herbal tea, which is rich in vitamins and minerals. Depending on the amount used, nettle tea has a mild to strong flavor and tastes similar to vegetable broth. Concentrated nettle tea can be used as a soup base or as a component in drinks or green cocktails. Nettle tea can also be used as a nutritional replacement for water. Nettle roots can be used as liquid or powdered extracts, as well as in special decoctions. Nettles are also used in herbal liquors [91]. In the British Isles, *Urtica* plants are used in an alcoholic beverage, which is similar to ginger beer and brewed in the same way. Nettle and oat extracts are the subject of a U.S. Patent describing the use of plant powders as additives in beverages or fruit juice to provide nutritional drinks [92]. Aqueous infusions of *U. dioica* exhibit antioxidant activity towards iron-promoted oxidation of phospholipids, linoleic acid and deoxyribose [83]. The use of such antioxidant and antimicrobial compounds is of considerable interest for the preservation of foods, as well as for improving the shelf-life of food products [53,93].

Despite their beneficial properties, the consumption of nettle teas or juices may cause a skin rash in individual cases. Although it is rare, there have been reports of allergic reactions after ingesting raw nettle leaves in the form of puree or juice [94]. Therefore, stinging nettles need to be correctly prepared by hot water infusion, maceration, drying or tincturing. This pretreatment deactivates the formic acid, allowing safe consumption of this valuable plant.

Oxidation is a serious problem in the food industry. Meat products are particularly susceptible to changes in the oxidation of lipids and heme pigments during storage. Along with meat spoilage bacteria, the oxidation of lipids and myoglobin has a major effect on reducing the shelf-life of meat and meat products. Lipid oxidation decreases the nutritional value of meat through the deterioration of essential fatty acids, causing an unacceptable flavor, generating potentially toxic products and promoting the oxidation of other important molecules, such as myoglobin [95]. Although synthetic antioxidants are available, in recent years, the demand for natural antioxidants has increased, mainly because of the adverse effects of synthetic antioxidants [96]. Iron, the most abundant ion in meat, is released from heme pigments and ferritin and may be an important catalyst in the oxidation of lipids and proteins. Studies have demonstrated that commercially-available polyphenols and extracts rich in polyphenols can inhibit myoglobin oxidation.

Inai et al. studied the inhibition of myoglobin oxidation by some plant polyphenols with activity for flavonols (kaempferol, myricetin and quercetin) [97]. Slightly weaker activity was observed for other polyphenols: sinapic acid, catechin, nordihydroguaiaretic acid, taxifolin, morin and ferulic acid. The use of natural antioxidants from *U. dioica* water extract and dried leaves as a functional ingredient significantly decreased the level of lipid deterioration and increased color stability during storage. Therefore, nettle water extract can be successfully used to reduce lipid oxidation and to enhance the functionality of the final products. Other studies have investigated the use of *U. dioica* water extract in sucuk, a Turkish dry-fermented sausage [98,99,100], in ground beef [101], meatballs [102], in super-chilled minced meat [103], vacuum-packed beef steaks [104] and in cooked pork sausage [105].

The use of *Urtica* spp. as a dietary supplement may positively affect the health and productivity of poultry and cows. The large number of active compounds in this plant may show stronger antibacterial activities than synthetic antimicrobials. It has been suggested that extracts of *Urtica* spp. may also possess appetite- and digestion-stimulating properties [106,107,108,109,110], which stimulate the growth of beneficial bacteria in the gastrointestinal tract of poultry [111]. In some parts of Europe, fresh leaves are traditionally fed to pigs and poultry. Safamehr and co-workers showed that nettles had no effect on the percentage of breast, thigh and abdominal fat [68]. However, chickens fed with 1% nettles had the highest carcass yield. In contrast, Alçiçek, Bozkurt and Çabuk observed an improvement in the carcass yield of broilers supplemented with a combination of essential oils [109]. This can be explained by more intense protein anabolism [112]. The relative weights of most organs were not affected by the inclusion of nettles to the diet, which is in agreement with the findings by Nobakht [113].

It has been suggested that the antioxidant activity of nettles may induce a decrease in the relative weight of the liver [114]. A study conducted by Safamehr and co-workers showed that 1%–2% supplementation with nettles had a positive effect on the performance, carcass traits and blood biochemical parameters of broilers [68]. A study by Humphries and Reynolds confirmed the usefulness of nettles as a forage crop for cows. Production of milk was maintained when nettles were used to replace dry grass silage in the diet of lactating dairy cows [115]. The addition of nettle haylage to the diet caused changes in rumen pH that were potentially beneficial to lactating dairy cows on high grain diets. It is worth noting that diets based on plant material, rich in immune-promoting bioactive compounds, can avoid the need for antibiotic growth promoters. There is increasing public and government pressure in several countries of the EU and some non-EU nations to find natural alternatives to antibiotics [116,117].

Şandru and co-workers noted that *U. dioica* alcoholic extract increased cell-mediated innate immune potential in chickens. Alcoholic nettle plant extract significantly increased total leukocyte numbers, from 15,400–17,125 cells/mm^3^ [118]. Similarly, nettle extract treatment significantly enhanced the in vitro functional capacity of phagocytes. This could lead to a higher resistance to diseases and improve the post-vaccination response of broilers, thus reducing economic losses. It was noted that the glucose and total protein content, as well as the heterophil and lymphocyte levels in chickens were not influenced by different quantities of nettle, while the concentrations of cholesterol and triglycerides in the blood were affected significantly. The effect on lowering cholesterol in blood serum may be because of the presence of plant sterols, such as stigmasterol and campesterol. These decrease the cholesterol concentration in micelles [119,120]. Fermont and co-workers and Visioli and co-workers report that the cholesterol levels in blood serum and meat are probably lowered by phenolic compounds [121,122]. However, Khosravi et al. found that the addition of nettle extract to the diet of boilers had no significant positive effect on their total cholesterol [123].

*Urtica* spp. provide animals with nutrients and bioactive components, which support antimicrobial activity, immune enhancement and stress reduction. However, the phytochemical composition is complex, and the mode of action is unclear [124]. Further studies are needed to investigate the bioactive components of nettles and their modes of action. It is worth noting that the World Health Organization (WHO), in its monographs on ‘Selected medicinal plants’, describes *Urticae* as valuable herbs for many medicinal uses [125]. The European Commission Directorate-General for Health and Food Safety showed that *Urtica* spp. fulfils the criteria of a foodstuff, as defined in Regulation (EC) No. 178/2002. This opinion is supported by the European Food Safety Authority (EFSA). It concluded that *Urtica* spp. has neither an immediate nor delayed harmful effect on human or animal health and has no negative effect on the environment [126].

## 6. Conclusions

Stinging nettles can be found all over the world. Plant hairs located on the leaves and stems contain a number of chemicals, which can cause a stinging reaction and uncomfortable irritation when brought into contact with human skin. Nevertheless, stinging nettles have a number of health benefits and have been used medicinally since at least the times of Ancient Greece. Studies have shown that all parts of the nettle have antioxidant, antimicrobial and pro-health capabilities. Most nettle medicines are made from the flowers, stems and leaves, but roots are also used in pharmacology. This valuable plant has been used most commonly as a diuretic and for treating painful muscles and joints, eczema, gout and anemia. Nettles may be used as a vegetable, in juice, tea and as an ingredient in many dishes. The use of *Urtica* spp. as a feed component could also positively affect the health of poultry and animal productivity. However, despite these proven benefits, the nettle is still an undervalued plant.

Research is continuing into this ordinary plant with unique pharmacological and dietary properties. It is worth investigating the possible wider inclusion of nettles in the daily diet to promote well-being and prevent diseases.

## Figures and Tables

**Table 1:** The list of species belonging to the *Urtica* genus [1,11,12].

No.	Name	Synonyms	Habitats
1	*U. angustifolia* Fisch. ex Hornem.	*U. dioica* var. *angustifolia* (Fisch. ex Hornem.) Ledeb. *U. foliosa* Blume	China, Japan, Korea
2	*U. ardens* Link	*U. himalayensis* Kunth & Bouché *U. mairei* var. *oblongifolia* C.J. Chen *U. zayuensis* C.J. Chen	Bhutan, India, Nepal, Sikkim
3	*U. atrichocaulis* (Hand.-Mazz.) C.J. Chen	*U. dioica* var. *atrichocaulis* Hand.-Mazz.	China -Guizhou, Sichuan, Yunnan
4	*U. atrovirens* Req. ex Loisel.	-	France, Italy, Spain
5	*U. ballotifolia* Wedd.	-	Colombia, Ecuador
6	*U. berteroana* Phil.	-	Chile, Bolivia, Argentina, Colombia
7	*U. buchtienii* Ross	-	Chile, Argentina
8	*U. cannabina* L.	*U. cannabina* f. *angustiloba* Chu	Russia, Sweden, Netherlands, China
9	*U. chamaedryoides* Pursh	*U. chamaedryoides* var. *runyonii* Correll	United States, Mexico
10	*U. circularis* Sorarú	*U. chamaedryoides* var. *circularis* Hauman *U. spathulata* var. *circularis* Hicken	Brazil, Argentina, Paraguay, Uruguay
11	*U. dioica* L.	*U. galeopsifolia* Wierzb. ex Opiz	United States, New Zealand, Turkey, Europe
12	*U. echinata* Benth.	*U. andicola* Wedd.	Bolivia, Peru, Argentina, Ecuador
13	*U. fissa* E. Pritz.	*U. pinfaensis* H. Lév. & Blin in H. Lév.	China, Taiwan, Egypt, Vietnam
14	*U. flabellata* Kunth	-	Bolivia, Peru, Ecuador, Chile, Colombia
15	*U. galeopsifolia* J. Jacq. ex Blume	-	Russia, Ukraine, Belarus
16	*U. glomeruliflora* Steud.	*U. fernandeziana* Ross ex Skottsb.	Chile
17	*U. haussknechtii* Boiss.	-	Turkey
18	*U. hyperborea* Jacq. ex Wedd.	*U. kunlunshanica* Chang Y. Yang	Nepal, India, China
19	*U. kioviensis* Rogow.	*U. dioica* var. *kioviensis* (Rogow.) Wedd. *U. dioica* subsp. *koviensis* Buia	Europe, Israel, Russia
20	*U. laetevirens* Maxim.	-	China, Japan, Korea
21	*U. leptophylla* Kunth	*U. copeyana* Killip *U. nicaraguensis* Liebm.	Costa Rica, Colombia, Peru, Bolivia, Ecuador
22	*U. lilloi* (Hauman) Geltman	*U. magellanica* var. *lilloi* Hauman	Argentina
23	*U. longispica* Killip	-	Ecuador, Peru, Colombia
24	*U. macbridei* Killip	-	Ecuador, Peru
25	*U. magellanica* Juss. ex Poir.	*U. bracteata* Steud. *U. darwinii* Hook. *U. dioica* var. *pycnantha* Wedd. & DC. *U. dioica* var. *steudelii* Wedd. *U. magellanica* subsp. *bracteata* (Steud.) Geltman *U. magellanica* var. *bracteata* (Steud.) Wedd. *U. pseudodioica* Steud.	Chile, Peru, Bolivia, Argentina, Ecuador
26	*U. mairei* H. Lév.	-	China, India, Bhutan
27	*U. masafuerae* Phil.	-	Chile
28	*U. membranacea* Poir. ex Savigny	*Dubrueilia membranacea* Gaudich. *U. caudata* Vahl *U. dubia* Forssk.	Europe, Algeria
29	*U. mexicana* Liebm.	-	Mexico, Guatemala
30	*U. mollis* Steud.	*U. dioica* var. *mollis* (Steud.) Wedd. *U. diplotricha* Phil.	Peru, Chile, Argentina
31	*U. morifolia* Poir.	-	Europe
32	*U. orizabae* Liebm.	-	Mexico, United States, Cuba
33	*U. parviflora* Roxb.	-	Nepal, India, United States, China, Bhutan
34	*U. pilulifera* L.	*U. dodartii* L.	Tunisia, Israel, Cyprus, Costa Rica, Turkey
35	*U. platyphylla* Wedd.	*U. dioica* subsp. *platyphylla* P. Medvedev *U. takedana* Ohwi	Japan, Russia
36	*U. praetermissa* V.W. Steinm.	-	Mexico
37	*U. pubescens* Ledeb.	-	Mexico
38	*U. rupestris* Guss.	-	Italy
39	*U. sondenii* (Simmons) Avrorin ex Geltman	*U. dioica* subsp. *sondenii* (Simmons) Hyl. *U. dioica* var. *sondenii* Simmons	Canada
40	*U. spiralis* Blume	-	Mexico
41	*U. stachyoides* Webb & Benth.	-	Spain, Mexico
42	*U. taiwaniana* S.S. Ying	-	Taiwan
43	*U. thunbergiana* Siebold & Zucc.	*U. macrorrhiza* Hand.-Mazz.	Japan, Korea, China
44	*U. triangularis* Hand.-Mazz.	-	China
45	*U. trichantha* (Wedd.) Acevedo & NAVAS	*U. echinata* var. *trichantha* Wedd.	Chile, Bolivia, Peru
46	*U. urens* L.	*U. trianae* Rusby	Unite States, Mexico, Europe, Israel, New Zealand

**Table 2:** Chemical composition of nettle leaf powders [10].

Parameter	Content
Moisture (%)	7.04 ± 0.77
Crude protein (%)	33.77 ± 0.35
Crude fiber (%)	9.08 ± 0.14
Crude fat (%)	3.55 ± 0.06
Total ash (%)	16.21 ± 0.54
Carbohydrate (%)	37.39 ± 0.72
Calcium (mg/100 g)	168.77 ± 1.47
Iron (mg/100 g)	227.89 ± 0.21
Tannins (%)	0.93 ± 0.01
Polyphenols (mg GAE/g)	128.75 ± 0.21
Carotenoids (μg/g, db)	3496.67 ± 0.56
Caloric value (kcal/100 g)	307.24 ± 0.13

**Table 3:** The phenolic profiles in *U. dioica* extracts (mg per g of dry extract) [20].

Group of Compounds	Compound	Origin							
		*Fruska Gora I/II*				*Stara Planina*			
		Flowers	Leaves	Steams	Roots	Flowers	Leaves	Steams	Roots
Phenolic acids	*p*-Hydroxybenzoic acid	0.064/0.017	0.037/0.021	0.021/0.023	0.032/0.029	0.036	0.051	0.014	0.048
	Gentisic acid	0.0096/0.0044	0.0034/0.0082	not det/det	not det/0.036	not det	det	not det	not det
	Protocatechuic acid	0.070/0.032	0.48/0.16	not det/0.014	not det/0.015	0.022	0.072	0.0069	0.0106
	Vanillic acid	det */not det **	not det/det	not det/not det	not det/det	not det	not det	not det	0.09
	**Quinic acid**	**1.6/0.27**	**0.3/0.36**	**0.047/0.039**	**0.1/0.31**	**0.86**	**0.66**	**0.088**	**0.36**
	Ferulic acid	0.071/0.09	0.009/0.013	0.031/0.061	0.011/0.028	0.05	0.052	0.024	0.024
	***p*** **-Coumaric acid**	**not det/0.0105**	**not det/not det**	**0.24/0.38**	**0.12/02**	**0.022**	**0.026**	**0.18**	**0.23**
	**Caffeic acid**	**0.48/0.41**	**0.21/0.29**	**0.0053/0.033**	**det/0.0118**	**0.64**	**0.93**	**0.031**	**0.0039**
	**5-*O*-Caffeolylquinic acid**	**36/15.8**	**1.23/2.7**	**0.29/1.87**	**0.056/0.029**	**35**	**28**	**2.3**	**0.025**
Coumarins	Esculetin	0.041/0.0078	0.0120/0.0125	0.015/0.019	det/0.0047	0.0095	0.0074	det	det
	Scopoletin	0.103/0.018	0.12/0.21	0.026/0.054	0.076/0.11	0.04	0.073	0.048	0.18
Lignans	Secoisolariciresinol	not det/not det	not det/not det	not det/not det	det/0.2	not det	not det	not det	0.009
Flavones	Chrysoeriol	det/det	det/det	det/det	det/det	0.0027	det	det	det
Flavonols	Kaempferol	det/0.007	not det/not det	not det/det	not det/not det	0.019	not det	not det	not det
	**Kaempferol 3-*O*-glucoside**	**0.074/0.7**	**not det/not det**	**not det/0.0068**	**not det/not det**	**0.6**	**0.07**	**0.017**	**not det**
	Quercitrin	0.0124/not det	not det/not det	not det/not det	not det/not det	not det	not det	not det	not det
	**Quercetin 3-*O*-glucoside**	**0.63/3.64**	**not det/0.0024**	**0.0316/0.38**	**not det/0.0054**	**2.82**	**1.08**	**0.48**	**not det**
	**Quercetin 3-*O*-rutinoside**	**6.1/4.6**	**0.0018/0.0206**	**0.40/1.35**	**0.0023/0.0186**	**9.5**	**4.6**	**2.25**	**0.0054**
	Isorhamnetin	det/0.036	not det/not det	not det/det	not det/not det	0.047	not det	not det	not det
Biflavonoids	Amentoflavone	det/det	det/det	det/det	det/det	det	det	det	det
Flavan-3-ols	Catechin	not det/0.076	not det/not det	not det/not det	not det/not det	1.0	not det	not det	not det

* det: peak observed, but the concentration was too low to evaluate it; ** not det: peak not observed. Bolded compounds are those that occur at the highest concentration.

**Table 4:** In vitro activity of *Urtica* spp. against microorganisms.

*Urtica* spp. Extract	Microorganisms	Location	Reference
*U. dioica* L. water extract	*Escherichia coli* *Proteus mirabilis* *Citrobacter koseri* *Staphylococcus aureus* *Streptococcus pneumoniae* *Enterobacter aerogenes* *Micrococcus luteus* *Staphylococcus epidermidis* *Candida albicans*	Turkey	[48]
*U. dioica* L. ethyl acetate extract	*Aeromonas hydrophila* *Salmonella typhi* *Staphylococcus aureus* *Bacillus cereus* *Escherichia coli*	Iraq	[28]
*U. dioica* L. water extract	*Salmonella* spp. *Proteus* spp. *Bacillus subtilis* *Staphylococcus aureus* *Pseudomonas aeruginosa* *Escherichia coli*	Iraq	[50]
*U. dioica* L. leaves aqueous extract	*Escherichia coli* *Enterococcus gallinarum* *Enterococcus faecalis* *Streptococcus pyogenes* *Clavibacter michiganensis* *Pseudomonas tomato* *Xanthomonas vesicatoria*	Turkey	[51]
*U. dioica* L. root aqueous extract	*Enterococcus faecalis* *Streptococcus pyogenes* *Klebsiella pneumoniae*		
*U. dioica* L. seeds aqueous extract	*Enterococcus faecalis*		
*U. dioica* L. aqueous extract 0.45 mg/100 mL	*Staphylococcus epidermidis* 3615 *Staphylococcus aureus* 740 *Escherichia coli* 443 *Salmonella typhimurium* 98 *Serratia marcescens* 97	India	[56]
*U. dioica* L. supercritical CO_2_ extract	*Bacillus subtilis* *Saccharomyces cerevisiae* *Aspergillus niger* *Botrytis cinerea* *Geotrichum candidum*	Macedonia	[49]
*U. dioica* L. ethanol extract	*Salmonella* spp. *Bacillus subtilis* *Staphylococcus aureus* *Pseudomonas aeruginosa*	Iraq	[50]
*U. dioica* L. methanol extract	15 different strains of *Staphylococcus aureus* MRSA	Iran	[54]
*U. dioica* L. methanol extract	*Shigella dysenteriae* *Salmonella enteritidis*	Iran	[55]
*U. dioica* L. leaves methanol extract	*Escherichia coli* *Streptococcus pyogenes* *Listeria monocytogenes* *Pseudomonas aeruginosa* *Klebsiella pneumoniae* *Proteus vulgaris* *Erwinia carotovora*	Turkey	[51]
*U. dioica* L. root methanol extract	*Enterococcus gallinarum* *Xanthomonas vesicatoria*		
*U. dioica* L. seeds methanol extract	*Enterococcus gallinarum* *Enterococcus faecalis* *Streptococcus pyogenes* *Staphylococcus aureus* *Listeria monocytogenes* *Pseudomonas aeruginosa* *Proteus vulgaris* *Shigella* spp. *Bacillus pumilus* *Clavibacter michiganensis* *Xanthomonas vesicatoria*		
*U. dioica* L. flowers methanol extract	*Escherichia coli* *Bacillus subtilis* *Staphylococcus aureus* *Pseudomonas aeruginosa* *Candida albicans* *Aspergillus niger*	Iran	[52]
*U. pilulifera* L. leaves aqueous extract	*Enterococcus faecalis* *Streptococcus pyogenes*	Turkey	[51]
*U. pilulifera* L. root aqueous extract	*Xanthomonas vesicatoria*		
*U. pilulifera* L. seeds aqueous extract	*Enterococcus faecalis* *Proteus vulgaris* *Shigella* spp.		
*U. pilulifera* L. leaves methanol extract	*Enterococcus gallinarum* *Enterococcus faecalis* *Streptococcus pyogenes* *Listeria monocytogenes* *Pseudomonas aeruginosa* *Klebsiella pneumoniae* *Proteus vulgaris* *Shigella* spp. *Bacillus pumilus* *Clavibacter michiganensis* *Pseudomonas tomato* *Erwinia carotovora*		
*U. pilulifera* L. roots methanol extract	*Enterococcus gallinarum* *Enterococcus faecalis* *Streptococcus pyogenes* *Clavibacter michiganensis* *Pseudomonas tomato* *Erwinia carotovora*		
*U. pilulifera* L. seeds methanol extract	*Enterococcus gallinarum* *Enterococcus faecalis* *Streptococcus pyogenes* *Staphylococcus aureus* *Listeria monocytogenes* *Pseudomonas aeruginosa* *Proteus vulgaris* *Shigella* spp. *Bacillus pumilus* *Clavibacter michiganensis*		
*U. urens* L. ethanol extract	*Staphylococcus aureus* ATCC 6538 *Pseudomonas aeruginosa* ATCC 27893 *Bacillus subtilis* JN 934392 *Salmonella enteritidis* *Escherichia coli* ATCC 25922 *Staphylococcus epidermidis* MTCC 3615 *Micrococcus luteus* ATCC 4698 *Enterococcus faecalis* ATCC 29212	Tunisia	[53]

**Table 5:** In vitro activity of *Urtica* spp. against microorganisms: minimal inhibitory concentrations.

*Urtica* spp. Extract	Microorganisms	Minimal Inhibitory Concentration (MIC)	Location	Reference
*U. dioica* L. ethanol extract	*Bacillus subtilis* *Escherichia coli* (food-origin) *Escherichia coli* (urine-origin) *Pseudomonas aeruginosa* *Lactobacillus plantarum*	36.21 mg/mL 36.21 mg/mL 72.43 mg/mL 72.43 mg/mL 72.43 mg/mL	Serbia	[16]
*U. dioica* L. hexane extract	*Staphylococcus aureus* MRSA *Bacillus cereus* *Bacillus spizizenii* ATCC 663 *Vibrio parahaemolyticus*	66.66 mg/mL 16.66 mg/mL 16.66 mg/mL 66.66 mg/mL	Iran	[57]
*U. dioica* L. chloroform extract	*Bacillus cereus* *Vibrio parahaemolyticus*	33.33 mg/mL 4.16 mg/mL		
*U. dioica* L.	*Acinetobacter calcoaceticus*	33.33 mg/mL		
*U. dioica* L. ethyl acetate extract I	*Bacillus spizizenii* ATCC 663 *Vibrio parahaemolyticus* *Saccharomyces cerevisiae*	8.33 mg/mL 16.66 mg/mL 2.08 mg/mL		
*U. dioica* L. ethyl acetate extract II	*Vibrio parahaemolyticus*	0.13 mg/mL		
*U. dioica* L. methanol extract	*Acinetobacter calcoaceticus*	16.66 mg/mL		
*U. dioica* L. butanol extract	*Escherichia coli* *Bacillus subtilis* *Staphylococcus aureus* MRSA	66.66 mg/mL 8.33 mg/mL 16.66 mg/mL		
*U. dioica* L. hexane fraction	*Aeromonas hydrophila* *Aeromonas salmonicida* subsp. *salmonicida* *Flavobacterium columnare* *Vibrio salmonicida* *Yersinia ruckeri* *Pseudomonas aeruginosa* *Staphylococcus aureus* *Salmonella typhi* *Klebsiella pneumoniae* *Enterococcus faecalis*	125 µg/mL 125 µg/mL 250 µg/mL 62.5 µg/mL 31.25 µg/mL 250 µg/mL 31.25 µg/mL 7.81 µg/mL 31.25 µg/mL 125 µg/mL	India	[58]
*U. dioica* L. leaves hexane fraction	*Staphylococcus aureus* *Pseudomonas aeruginosa* *Salmonella typhi* *Klebsiella pneumoniae* *Shigella flexneri*	31.25 µg/mL 250 µg/mL 7.81 µg/mL 31.25 µg/mL 125 µg/mL	India	[59]
*U dioica* L. essential oils hydrodistillation method	*Bacillus cereus* PTCC1565 *Staphylococcus aureus* *Pseudomonas aeruginosa* *Klebsiella pneumoniae* *Enterococcus faecalis* PTCC1239 *Escherichia coli* ATCC1533	1.8 µg/mL 3.75 µg/mL 3.75 µg/mL 3.75 µg/mL 7.5 µg/mL 7.5 µg/mL	Iran	[60]
*U. dioica* L. ethanol extract	Methicillin-sensitive strains of *S. aureus* Methicillin-resistant strains of *S. aureus*	0.188–0.500 mg/mL 0.063–0.500 mg/mL	Portugal	[61]
